# Transactions of the Twentieth Anniversary Meeting of the Illinois State Medical Society

**Published:** 1871-01

**Authors:** 


Transactions of the Twentieth Anniversary Meeting of the Illinois State Medical Society, held in Dixon, May 17–18, 1870.

The Transactions, though late in distribution to members, have been published in good style, and make a volume of 141 pages. The volume contains a pretty full record of the proceedings of the Anniversary Meeting, with the following reports and papers: Report on Practical Medicine, a paper by James S. Whitmire, M.D., of Metamora, and one on Blood-letting Not Necessary in Pneumonia, by D. W. Young, M.D., of Aurora; Report on Surgery, by Moses Gunn, M.D., of Chicago; On Drugs and Medicines, by Chas. Hunt, M.D., of Dixon; On Ophthalmology, by E. L. Holmes, M.D., of Chicago; On Otology, by S. J. Jones, M.D., of Chicago; On Statistics of Diseases of the Ear, by E. L. Holmes, M.D., of Chicago; On the Use of Plaster of Paris in Fractures, by R. E. Bogue, M.D., of Chicago; Amputation at the Hip-joint, for Disease of the Hip and Knee-joints, by E. Powell, M.D., of Chicago; On the Communication from the American Medical Association, in Relation to Licenses to Practice, by N. S. Davis and D. Prince, Special Committee; List of Members; Constitution and By-Laws of the Society; and Code of Ethics. Several of the reports have been published in the *Examiner*, but any reader will find enough new matter to fully remunerate him for obtaining a copy of the Transactions in full.

Any information concerning the volume may be obtained by addressing the Permanent-Secretary, Dr. T. D. Fitch, M.D., of Chicago.

